# Correction: Association of Rare Genetic Variants in Opioid Receptors with Tourette Syndrome

**DOI:** 10.5334/tohm.792

**Published:** 2023-07-14

**Authors:** Christel Depienne, Sorana Ciura, Oriane Trouillard, Delphine Bouteiller, Elsa Leitão, Caroline Nava, Boris Keren, Yannick Marie, Justine Guegan, Sylvie Forlani, Alexis Brice, Mathieu Anheim, Yves Agid, Paul Krack, Philippe Damier, François Viallet, Jean-Luc Houeto, Franck Durif, Marie Vidailhet, Yulia Worbe, Emmanuel Roze, Edor Kabashi, Andreas Hartmann

**Affiliations:** 1 INSERM, U 1127, CNRS UMR 7225, Faculté de Médecine de Sorbonne Université, UMR S 1127, Institut du Cerveau et de la Moelle épinière, ICM, Hôpital Pitié-Salpêtrière, 47-83 Boulevard de l’Hôpital, 75013 Paris, FR; 2 Institute of Human Genetics, University Hospital Essen, University of Duisburg-Essen, 45122 Essen, DE; 3 Assistance Publique Hôpitaux de Paris (AP-HP), Hôpital Pitié-Salpêtrière, Département de Génétique, 47-83 Boulevard de l’Hôpital, 75013 Paris, FR; 4 Service de neurologie, CHU de Strasbourg, Hôpital de Hautepierre, Avenue Molière, 67200 Strasbourg Strasbourg, FR; 5 Service de Neurologie, CHU de Grenoble, Avenue Maquis du Grésivaudan, 38700 La Tronche, FR; 6 Center for Movement Disorders, Inselspital, University of Bern, Freiburgstrasse 18, 3010 Bern, Switzerland; 7 Service de Neurologie, CHU de Nantes, 5 Allée de l’Île Gloriette, 44093 Nantes, FR; 8 Service de Neurologie, CRHU d’Aix-en-Provence, Avenue des Tamaris, 13100 Aix-en-Provence, FR; 9 Service de Neurologie, CHU de Poitiers, 2 Rue de la Milétrie, 86021 Poitiers, FR; 10 Service de Neurologie, CHU de Clermont-Ferrand, CHU de Clermont-Ferrand, Hôpital Gabriel Montpied, 58 rue Montalembert, 63003 Clermont-Ferrand, FR; 11 Assistance Publique Hôpitaux de Paris (APHP), Hôpital Pitié-Salpêtrière, Département de Neurologie, 47-83 Boulevard de l’Hôpital, 75013 Paris, FR; 12 AP-HP, Centre de Référence National Maladie Rare ‘Syndrome Gilles de la Tourette’, Hôpital Pitié- Salpêtrière, 47-83 Boulevard de l’Hôpital, 75013 Paris, FR

**Keywords:** Tourette syndrome, gene, variant, susceptibility factor, opioid receptor, zebrafish, OPRK1

## Abstract

This article details a correction to: Depienne C, Ciura S, Trouillard O, Bouteiller D, Leitão E, Nava C, et al. Association of Rare Genetic Variants in Opioid Receptors with Tourette Syndrome. *Tremor and Other Hyperkinetic Movements*: 2019; 9(0). DOI: https://doi.org/10.5334/tohm.464.

## Correction

This is a correction to the article “Association of Rare Genetic Variants in Opioid Receptors with Tourette Syndrome” [1]. The acknowledgments section of the original article should have included the following acknowledgment:

“The authors thank the French Association for Tourette Syndrome (AFSGT) for their generous financial support”.

This correction was brought to the attention of the journal by the author Andreas Hartmann.

## Competing interests

The authors have no competing interests to declare.
